# Effect of dietary mannanoligosaccharide supplementation on nutrient digestibility, hindgut fermentation, immune response and antioxidant indices in dogs

**DOI:** 10.1186/s40781-017-0136-6

**Published:** 2017-05-11

**Authors:** Mahesh M. Pawar, Ashok K. Pattanaik, Dharmendra K. Sinha, Tapas K. Goswami, Kusumakar Sharma

**Affiliations:** 1 Division of Animal Nutrition, ICAR-Indian Veterinary Research Institute, Izatnagar, 243122 India; 2 Present address: Department of Animal Nutrition, College of Veterinary Science & Animal Husbandry, Sardarkrushinagar Dantiwada Agricultural University, Sardarkrushinagar, 385506 India; 3 Present address: Carl R. Woese Institute for Genomic Biology, University of Illinois at Urbana-Champaign, Urbana, IL 61801 USA; 4 Division of Epidemiology, ICAR-Indian Veterinary Research Institute, Izatnagar, 243122 India; 5 Immunology Section, ICAR-Indian Veterinary Research Institute, Izatnagar, 243122 India

**Keywords:** Antioxidants, Dogs, Hindgut fermentation, Immunity, Mannanoligosaccharide

## Abstract

**Background:**

Use of prebiotics in companion animal nutrition is often considered advantageous over probiotics because of the ease of handling, ability to withstand processing and storage etc. While most of the studies on prebiotic use in dogs have been done with processed food as basal diet, the response in relation to homemade diet feeding is not very well explored.

**Methods:**

The study was conducted to evaluate the effects of dietary mannanoligosaccharide (MOS) supplementation on nutrient digestibility, hindgut fermentation, immune response and antioxidant indices in dogs. Ten Spitz pups were divided into two groups: control (CON) with no supplementation, and experimental (MOS) wherein the basal diet was supplemented with MOS at 15 g/kg diet. All dogs were fed on a home-prepared diet for a period of 150 days. The study protocol included a digestion trial, periodic blood collection and analysis for lipid profile and erythrocytic antioxidants. Immune response of the animals was assessed towards the end of the feeding period.

**Results:**

Results revealed no significant (*P > 0.05*) variations in palatability score, intake and apparent digestibility of nutrients between the groups. Faecal score, faeces voided, faecal pH, concentrations of ammonia, lactate and short-chain fatty acids were comparable (*P > 0.05*) between the two groups. Cell-mediated immune response, assessed as delayed-type of hypersensitivity response, was significantly higher (*P < 0.05*) in the MOS group. The percent of lymphocyte sub-populations CD4+ and ratio of CD4+:CD8+ were also significantly (*P < 0.05*) higher in MOS group. The serum IgG levels were similar (*P > 0.05*) in both the groups. Supplementation of MOS lowered (*P < 0.05*) serum total- and LDL- cholesterol levels, when compared with the control group. The erythrocytic antioxidant indices were similar (*P > 0.05*) between the two groups.

**Conclusions:**

The results indicated that supplementation of MOS at the rate of 15 g/kg in the diet of dog augmented the cell-mediated immune response and serum lipid profile without any influences on digestibility of nutrients, hindgut fermentation and antioxidants indices.

## Background

A nutritionally balanced diet and proper microbial ecology are required for a healthy gut. Optimization of gut health through feeding of probiotics that help maintaining the microbial balance of hindgut has become a routine approach in human and animal nutrition. However, the difficulty in maintaining the liveability and therefore the effectiveness of the probiotic bacteria in varied processing and management conditions has been a concern in canine nutrition practice. In this scenario, prebiotics are often considered to have advantages over the probiotics. Prebiotics are defined as selectively fermented ingredients that allow specific changes, both in the composition and/or activity in the gastrointestinal microflora that confers benefits upon host well-being and health [1]. Prebiotics have been demonstrated to possess the potential to improve or maintain a balanced intestinal microflora resulting in enhanced health and wellbeing of animals. Mannanoligosaccharide (MOS) derived from the outer cell wall of yeast has been used as a potential prebiotic to improve health status in many species [2, 3] including dogs [4]. It improves gut microflora balance and has immune-modulatory properties [5]. Previous published reports suggest that dietary supplementation of MOS has positive influence on the immune system of dogs [6–8].

The role of MOS in improving lipid profile by lowering serum concentrations of cholesterol, LDL-cholesterol, and triglyceride have been proposed in human [9] and animals [10]. Nutrient mobilization and changing digestive efficiency during active growing phase lead to inflammatory responses and production of reactive oxygen species (ROS) that may influence the antioxidant status. Several enzymatic systems are found in tissues to prevent oxidative damage by quenching ROS. These include antioxidants like superoxide dismutase, reduced glutathione, catalase, lipid peroxidation, glutathione peroxidase and total thiol groups [11]. However, the effects of dietary MOS supplementation on lipid profile and antioxidant indices have not yet been investigated in dogs. Likewise, most of the studies on prebiotics including MOS have been carried out using dogs fed processed pet foods. However, studies on the effects of dietary MOS on the digestive and fermentative attributes of dogs fed on home-prepared diet are still lacking. This aspect is important as in many situations pet dogs are reared on homemade diets because of behavioural, medical and socio-economic reasons. Therefore, the aim of this study was to evaluate the effects of dietary MOS supplementation on nutrient digestibility, hindgut fermentation, immune response, lipid profile and antioxidant indices in dogs when fed on a home cooked diet.

## Methods

The study was carried out in the kennel facility of Animal Nutrition Division, Indian Veterinary Research Institute, Izatnagar, India.

### Experimental animals and dietary treatments

Ten Spitz pups (age 4 months; average body weight 4.2 kg) were divided into two equal groups: control (CON) and experimental (MOS), and fed a home-prepared diet (Table 1) as per the NRC [12] recommendations for 150 days. The MOS group was supplemented with MOS powder (derived from the cell wall of *Saccharomyces cerevisiae;* containing 300 g protein,14 g crude fat, and 130 g crude fibre per kg; courtesy, Provimi Animal Nutrition India Pvt. Ltd., Bangalore, India) at the rate of 15 g/kg of food. The measured quantity of MOS was top-dressed on weighed amount of food for individual dogs and mixed thoroughly. The total quantity of food required by individual dog was offered in clean feeding bowls in two equal portions in morning (09:30) and evening (19:30). The dogs had access to drinking water throughout the day. A 1–4-point scale was used for assessment of palatability of the experimental diets [13]. The experimental protocol was approved by the Institutional Animal Ethics Committee (IAEC), Indian Veterinary Research Institute, Izatnagar, India.

**Table 1:** Ingredient and chemical composition of experimental diet fed to the dogs^a^

Ingredients	(g/kg diet)	Chemical composition	(%)
Rice	450.0	Organic matter	96.2
Extruded soya	474.0	Crude protein	22.4
Soya oil	50.0	Crude fat	7.30
Iodized salt	3.0	Crude fibre	4.67
Dicalcium phosphate	13.0	Total ash	3.84
Calcium carbonate	10.0	Nitrogen-free extract	61.8
Trace mineral premix^b^	1.0	Calcium	1.02
Vitamin premix^c^	1.0	Phosphorus	0.81

^a^Pressure cooked at 15 psi for 10 min, Metabolizable energy = 3.56 kcal/g

^b^Provided (per kg diet): Mn 14.2 mg; Fe 110 mg; Cu 9 mg; Co 1.8 mg; Zn 150 mg; I 1.6 mg; Se 0.3 mg

^c^Provided (per kg diet): vitamin A 10,000 IU; vitamin D 1000 IU; vitamin E 100 IU; vitamin K 0.65 mg; thiamin 7.56 mg; riboflavin 11.89 mg; pantothenic acid 18.50 mg; niacin 93.16 mg; pyridoxine 6.60 mg; biotin 12.42 mg; folic acid 1142.10 μg; vitamin B_12_ 164.87 μg

### Digestion trial and hindgut fermentation

A digestion trial of 4-days duration was conducted after 60 days of experimental feeding. It involved quantification of daily food intake and faecal excretion to assess the digestibility of nutrients. The faecal score was recorded using a 1–5-point scale [14]. The pooled fresh faecal samples were mixed thoroughly and an aliquot was obtained for nitrogen estimation, which was preserved in 1:4 dilute H_2_SO_4_. Another aliquot of one gram of fresh faeces was diluted with an equal volume of distilled water and centrifuged at 10,000 rpm. The supernatant was used for determining pH, and ammonia and lactate. A third aliquot of one gram of fresh faeces was mixed with 1 mL of 25% of metaphosphoric acid and centrifuged at 10,000 rpm for 10 min. The supernatant was used for short-chain fatty acids (SCFAs) analysis. A final aliquot was obtained from rest of the fresh faecal samples for determination of DM. The samples of faeces and food were dried at 60 °C in a forced-draft oven, ground through a 2-mm screen in a laboratory mill (SM100, Retsch GmbH, Haan, Germany) and used for further analysis. The ground samples of food offered and faeces were analyzed in triplicate for DM, crude protein (CP), crude fat, crude fibre (CF), ash, calcium and phosphorus according to the procedures of AOAC [15]. Nitrogen-free extract (NFE) was calculated by difference. The pH of faecal samples was measured by using a pH meter (Eutech Instruments, Malaysia). Faecal ammonia [16] and lactate [17] concentration were estimated adopting standard methods. The SCFAs concentrations were estimated by using gas–liquid chromatography (Neucon-5765) equipped with an FID and Chromosorb 101 glass column (4 ft. length and 1.8 mm diameter) as described by Agarwal et al. [18].

### Immune response

The cell-mediated immune (CMI) response was assessed at 110 day of the study by measuring the skin induration as delayed-type hypersensitivity (DTH) reaction to intra-dermal inoculation of phytohaemagglutinin-P (PHA-P) mitogen as detailed elsewhere [19, 20]. Additionally, whole blood samples were collected into chilled heparinized micro-centrifuge tubes, and were utilized for immune phenotyping of T-lymphocyte subpopulations by flow cytometry. Commercially available monoclonal antibodies (Serotec, Oxford, UK) were used to identify cell surface markers for T-cells CD3, CD4+ and CD8+. The lymphocyte subsets were quantified by using dual-laser bench-top FACScan flow cytometer (FACSCalibur, Becton Dickinson, San Diego, CA, USA) as described previously [20]. At 120 day of the study, humoral immune (HI) response of the dogs was assessed by measuring serum levels of immunoglobulin G (IgG) following subcutaneous inoculation of Leptospira antigen (Intervet India Limited, Pune, India). The blood samples were collected at 0, 7, 14 and 28 days post-inoculation by taking aseptic precautions and centrifuged at 2000 rpm for 20 min for the separation of serum. Serum levels of IgG were measured using single radial immuno-diffusion test kit (VMRD Inc., Pullman, WA). Briefly, 3 μL of the canine IgG standard at concentrations of 300, 600, 1200 and 2400 mg/dl were added to wells on the plate in duplicate. The plates were incubated in a humidified chamber for 48–72 h at room temperature until the size of the precipitin ring was stable. Using a centimeter ruler, the ring diameter was measured and standard curve was generated. The serum samples (diluted 1:100) were assayed in duplicate using the same procedure.

### Antioxidant indices and lipid profile

The blood samples were collected at 120-day of experimental feeding into sterilized micro-centrifuge tubes containing acid citrate dextrose for analysis of antioxidants. Blood samples were centrifuged at 3000 rpm for 20 min and sedimented cells were washed with 0.9% NaCl solution and re-centrifuged three times with phosphate buffer saline. The washed erythrocytes were then haemolysed with nine volumes of distilled water to prepare 10% erythrocytic haemolysate. Estimation of enzymatic-﻿ (superoxide dismutase: SOD and catalase) and non-enzymatic- (lipid peroxidation: LPO, reduced glutathione: GSH and total thiols) antioxidant indices in the erythrocytic haemolysate were carried out as described earlier [21]. The blood samples collected were analyzed in duplicate to determine serum concentrations of triglyceride, total cholesterol and high-density lipoprotein (HDL)-cholesterol using diagnostic kits (Span Diagnostics Limited, Surat, India). The low-density lipoprotein (LDL; LDL = Total cholesterol–HDL – triglycerides/5) and very low-density lipoprotein (VLDL; VLDL = triglycerides/5) were calculated.

### Statistical analysis

All the experimental data obtained were statistically analyzed by using SPSS v.16.0 (SPSS Inc., Chicago IL) as per the standard statistical methods [22]. Significant differences between means of treatments were assessed by the Duncan’s test, and the differences among treatments were declared significant at *P < 0.05*.

## Results and discussion

### Nutrient digestibility and body weight changes

The palatability score, intake of DM, CP, fat, CF and NFE were comparable between the groups (Table 2). Similarly, apparent digestibility of DM, fat, CF and NFE did not differ (*P > 0.05*) between the groups. The CP digestibility, however, tended (*P > 0.05*) to be lower in MOS supplemented group as compared to CON group. It is reported that dietary supplementation of oligosaccharides at various dose levels did not affect apparent total-tract digestibility of DM, CP and fat of dogs [4, 23]. However, other studies with dogs found that oligosaccharides supplementation decreased total tract digestibility of DM, OM, and CP [10, 24]. Prebiotics are added to the diet to facilitate changes in the microbial micro-climate of the hind gut. Most of digestion process is completed by the time the digesta reaches the colon of the dog. Hence, it is hardly expected that dietary supplementation of prebiotics would have worthwhile impact on the digestibility of nutrients. This could possibly the reason why no positive impact of the MOS was evident on the digestibility of nutrients. In general, when the diet is high in protein or it contains poorly digestible protein ingredients, the undigested proteins reaching the colon get converted to ammonia because of increased proteolysis. Supplementation of prebiotics in these situations may reduce ammonia production because of the ensuing higher fermentation, leading to increased synthesis of bacterial proteins [25]. This, in turn, may result in a lowering of apparent digestibility of CP because the bacterial proteins so produced are not digested/absorbed at colonic level, and are excreted in the faeces. This could also explain the trend of lower CP digestibility observed in the MOS group in the present study.

**Table 2:** Effects of dietary mannanoligosaccharide supplementation on the total tract apparent digestibility of nutrients and body weight gain of dogs

Attributes	Dietary groups		Significance
	CON	MOS	
Palatability score^a^	1.43 ± 0.11	1.50 ± 0.19	NS
Digestibility (%)			
Dry matter	82.1 ± 1.38	83.6 ± 0.63	NS
Crude protein	80.0 ± 2.06	76.1 ± 3.62	NS
Crude fat	90.9 ± 2.79	91.9 ± 1.31	NS
Crude fibre	51.7 ± 1.26	55.2 ± 0.82	NS
Nitrogen free extract	79.9 ± 1.82	81.1 ± 0.64	NS
Body weight changes (kg)			
Initial	4.31 ± 0.33	4.27 ± 0.38	NS
Final	6.07 ± 0.32	6.11 ± 0.42	NS
Net gain	1.76 ± 0.55	1.84 ± 0.32	NS

*CON* control diet, *MOS* control diet + mannanoligosaccharide at 15 g/kg diet

^a^Based on 1–4 point scale

*NS* non-significant; *P > 0.05*

In this study, feeding the dogs with 15 g MOS/kg of food did not affect body weight (Table 2). This could be due to the fact that the animals used were already adults by the time the experiment was completed﻿,﻿ and were fed on the same basal diet with the addition of only 1.5% of MOS. Like the present findings, O’Carra [6] reported no difference in body weight gain of Border Collie pups fed diet supplemented with 0.2% dietary MOS.

### Hindgut fermentation

The faecal score, weight of faeces voided and faecal DM were without any significant (*P > 0.05*) difference between the two groups (Table 3). In addition, MOS supplementation did not affect (*P > 0.05*) faecal pH, faecal concentrations of ammonia and lactate. Faecal concentrations of acetate, propionate, butyrate and total SCFAs were comparable (*P > 0.05*) in both the groups. In line with the present observations, Diez et al. [26] also did not find any increase in faecal volume of dogs fed diet supplemented with inulin (6.5%) and oligofructose (5%). Similarly, Flickinger et al. [27] reported that wet faecal output, faecal score, faecal pH and ammonia concentrations were unaffected in dogs supplemented with oligofructose at the level of 1 g/d. In contrast to the present findings, Propst et al. [24] found concentrations of faecal acetate, propionate, butyrate and total SCFAs were higher (*P* < 0.01) in dogs supplemented with oligofructose (0.3, 0.6 and 0.9% on DM basis) than in control. Swanson et al. [7] observed the similar increase in SCFAs concentrations in dogs supplemented with oligosaccharides. The reported variations observed in the fermentative metabolites could be due to various factors related to the dose and nature of the prebiotics as well as the composition of the basal diet [28]. In the present study, the dietary level of MOS used was 1.5% which is equivalent to a daily intake of 3.26 g per dog, while most of the studies have used MOS at much lower levels [7, 25, 29]. However, even at the stated level of supplementation, the faecal concentrations of acetate and butyrate were 11.7 and 12.4% higher in comparison to the respective CON group values. Nonetheless, a potential factor preventing the detection of differences in faecal SCFA concentration between the two groups in the current study could possibly the rapid absorption of SCFA by colonocytes, as has been suggested by von Englehardt et al. [30], and endorsed by Swanson et al. [7].

**Table 3:** Effects of dietary mannanoligosaccharide supplementation on faecal fermentative attributes of dogs

Attributes	Dietary groups		Significance
	CON	MOS	
Physical attributes			
Faecal score^a^	3.33 ± 0.33	3.60 ± 0.40	NS
Faeces voided (g/day)	156.8 ± 14.8	164.9 ± 14.3	NS
Faecal DM (g/kg)	218.6 ± 21.8	216.6 ± 18.4	NS
Chemical attributes			
pH	5.84 ± 0.23	5.77 ± 0.21	NS
Ammonia (μmol/g)	8.45 ± 0.87	8.39 ± 1.24	NS
Lactate (μmol/g)	24.25 ± 2.07	27.85 ± 5.15	NS
Acetate (μmol/g)	134.1 ± 6.12	146.5 ± 11.4	NS
Propionate (μmol/g)	100.4 ± 12.0	97.13 ± 6.77	NS
Butyrate (μmol/g)	31.46 ± 3.04	35.40 ± 1.88	NS
Total SCFAs (μmol/g)	265.9 ± 14.9	278.9 ± 16.2	NS

*CON* control diet, *MOS* control diet + mannanoligosaccharide at 15 g/kg diet

^a^Based on 1–5 point scale

*NS* non-significant; *P > 0.05*, *SCFAs* short-chain fatty acids

### Immune response

**Fig. 1:**
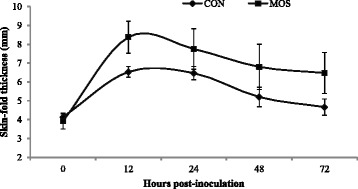
Effect of dietary mannanoligosaccharide on cell-mediated immune response assessed as increase in skin-fold thickness (mm) following intra-dermal inoculation of phytohaemagglutinin-P mitogen in dogs. CON, control diet; MOS, control diet supplemented with mannanoligosaccharide at 15 g/kg diet. (Significance: Diet, *P = 0.004*; Hour, *P < 0.001*; Diet × Hour, *P = 0.480*)

The MOS and the immune system interaction was expected as mannans and glucans found in cell walls of *S. cerevisiae* have been shown to induce an antigenic response [31], and modulate immunity due to the direct influence of the MOS on immune system and/or improved intestinal absorption of some nutrients, such as zinc, copper, selenium [32]. The DTH reaction is a good indicator of events occurring at the effector phase of the CMI response in vivo [19]. The DTH response in terms of skin thickness increment was significantly higher (*P < 0.05*) in the MOS group, when compared with the CON group (Fig. 1). The highest skin induration was noted at 12 h of post-inoculation in both the groups. The population of lymphocyte subpopulations CD3 and CD8+ were similar (*P > 0.05*) in both the groups. However, the population of CD4+ (43.7 vs. 45.6) and ratio of CD4+:CD8+ (2.5 vs. 2.9) were significantly higher (*P < 0.05*) in the MOS group than in the CON group (Table 4). CD4+ lymphocytes are helper–inducer T-lymphocytes, whereas CD8+ cells are suppressor or cytotoxic T-cells. T-helper CD4+ is a humoral immune response indicator, which help other white blood cells in immunologic processes. The CD4+ interferes in the stimulation and maturation of B lymphocytes and indicates how well the immune system is working [33]. The significant increase in population of CD4+ T-lymphocytes in the present study may therefore indicate positive influence of MOS on the immune system. This may also explain the enhanced DTH response observed in MOS group as compared to CON group. Because CD4+ T-cells play an important role in inducing the DTH response and activated CD4+ T-cells are potent source of various cytokines. The CD4+:CD8+ ratio is another important indicator of immune status [34], and a lower ratio is indicative of reduced immune status. A decrease in CD4+ and a CD4+:CD8+ ratio of less than 1.5 has been correlated with immune impairment and increased susceptibility to infection [35]. The greater ratio of CD4+:CD8+ in MOS group indicates improved systemic immune response in the experimental group of dogs induced by MOS as compared to CON group. There have been reports of alterations of the CD4+ and CD8+ T cell proportions in the gut-associated lymphatic tissue in canines in response to fermentable fibres [36]. Samal et al. [13] have also reported improved CD4+ population and CD4+:CD8+ ratio in dogs supplemented with graded levels of Jerusalem artichoke as a source of prebiotic.

**Table 4:** Effect of dietary mannanoligosaccharide supplementation on peripheral lymphocyte subpopulations of dogs

Lymphocytes (%)	Dietary groups		Significance
	CON	MOS	
CD3	81.73 ± 1.07	82.04 ± 0.97	NS
CD4+	43.70^a^ ± 0.29	45.64^b^ ± 0.20	*
CD8+	17.20 ± 0.31	16.00 ± 0.22	NS
CD4+:CD8+	2.54^a^ ± 0.06	2.85^b^ ± 0.05	*

*CON* control diet, *MOS* control diet + mannanoligosaccharide at 15 g/kg diet

^ab^Means bearing different superscripts in a row differ significantly; *P < 0.05, *NS* non-significant; *P > 0.05*

**Fig. 2:**
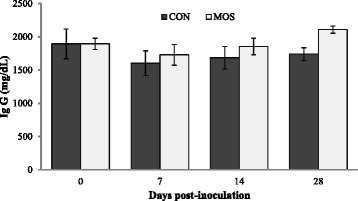
Effect of dietary mannanoligosaccharide on serum IgG levels of dogs assessed by single radial immunodiffusion test. CON, control diet; MOS, control diet supplemented with mannanoligosaccharide at 15 g/kg diet. (Significance: Diet, *P = 0.084*; Day, *P = 0.160*; Diet × Day, *P = 0.899*)

The serum IgG levels at 0, 7, 14 and 28 days of post-inoculation with Laptospira antigen were without any significant (*P > 0.05*) differences (Fig. 2). However, dietary MOS supplementation tended to increase (*P = 0.084*) the serum levels of IgG in MOS (1956 ± 67.1 mg/dL) group of dogs in comparison to the CON (1731 ± 84.1 mg/dL) group. This, when interpreted in conjunction with the observed increase in CD4+ population, indicates that dietary MOS supplementation might have had a stimulating influence on the humoral immune response of the dogs. Contrary to the present findings, O’Carra [6] reported no change plasma IgG levels in Border Collie pups and adult Beagles fed diet supplemented, respectively, with 2 g and 1-4 g MOS/kg. However, the differences between these reported studies and our results could be explained by the lower doses of MOS used in their experiments.

### Antioxidant indices and lipid profile

The data on erythrocytic antioxidants indicated no influence of MOS on the antioxidant enzymes viz. SOD, and catalase. The extent of lipid peroxidation, and the concentrations of GSH and total thiols also remained uninfluenced (Table 5). There have been studies indicating that mannans from *S. cerevisiae* have antioxidative property in vitro [37]. Ognik and Krauze [38] have reported that dietary use of MOS in turkey stimulated the mechanisms of oxidative defense and improved the growth performance of the birds. However, no influence of MOS supplementation was evident on the measured antioxidants in the present study. It would be reasonable to construe that the dogs in the present case were devoid of any stress, and hence no differences could be seen in the antioxidant status between the two groups of dogs.

**Table 5:** Effects of dietary mannanoligosaccharide supplementation on lipid profile and erythrocytic antioxidant indices of dogs

Attributes	Dietary groups		Significance
	CON	MOS	
Lipid profile			
Triglycerides (mg/dL)	49.31 ± 4.10	47.09 ± 5.91	NS
Total cholesterol (mg/dL)	139.83^b^ ± 4.14	125.38^a^ ± 4.31	*
HDL-cholesterol (mg/dL)	63.42 ± 5.73	69.63 ± 5.63	NS
LDL-cholesterol (mg/dL)	66.63^b^ ± 6.16	46.33^a^ ± 5.01	*
VLDL-cholesterol (mg/dL)	9.78 ± 0.79	9.42 ± 1.18	NS
Antioxidant indices			
SOD (U/mg Hb)	54.28 ± 3.07	57.38 ± 3.38	NS
Catalase (U/mg Hb)	0.150 ± 0.01	0.146 ± 0.02	NS
LPO (nmoL MDA/mg Hb)	2.59 ± 0.24	2.45 ± 0.26	NS
GSH (nmoL/mg Hb)	2.48 ± 0.11	2.61 ± 0.17	NS
Total thiols (nmoL/mg Hb)	1.83 ± 0.07	1.78 ± 0.07	NS

*CON* control diet, *MOS* control diet + mannanoligosaccharide at 15 g/kg diet

*HDL* high-density lipoproteins, *LDL* low-density lipoproteins, *VLDL* very low-density lipoproteins, *SOD* superoxide dismutase, *LPO* lipid peroxidation, *MDA* malondialdehyde, *GSH* reduced glutathione, *Hb* haemoglobin

^ab^Means bearing different superscripts in a row differ significantly; *P < 0.05, *NS* non-significant; *P > 0.05*

Supplementation of MOS in diet of dogs significantly (*P < 0.05*) reduced serum total- (125.4 vs. 139.8 mg/dL) and LDL- cholesterol (46.3 vs. 66.6 mg/dl) concentrations, when compared to the CON group (Table 5). It has been suggested that the products of bacterial fermentation, specifically SCFAs, may inhibit cholesterol synthesis in the liver and/or cause the mobilization of plasma cholesterol to the liver [39] leading, in turn, to a possible reduction in cholesterol in blood plasma. The observed lowered levels total- and LDL- cholesterol in the present are similar to the reports of Diez et al. [8] who found that dietary oligofructose supplementation (5% and 10.2%) in dog diets tended (*P > 0.05*) to lower plasma cholesterol levels, when compared with the control diet (146 and 144 vs. 157 mg/dl, respectively). On the other hand, serum levels of triglycerides, HDL- and VLDL- cholesterol were comparable (*P > 0.05*) in both the groups.

## Conclusions

The supplementation of MOS at 15 g/kg diet of dogs did not have any influence on the digestibility of nutrients, hindgut fermentation and the erythrocytic antioxidant indices. The MOS supplementation, however, improved the immune status and lipid profile of dogs. Further studies, however, are warranted to establish the positive role of MOS with varied supplemental levels.

## Abbreviations

CF: Crude fiber
CMI: Cell-mediated immunity
CP: Crude protein
DM: Dry matter
DTH: Delayed-type hypersensitivity
GSH: Reduced glutathione
HDL: High-density lipoproteins
IgG: Immunoglobulin G
LDL: Low-density lipoproteins
LPO: Lipid peroxidation
MOS: Mannanoligosaccharides
NFE: Nitrogen-free extract
PHA-P: Phytohaemagglutinin-P
ROS: Reactive oxygen species
SCFA: Short-chain fatty acids
SOD: Superoxide dismutase
VLDL: Very low-density lipoproteins
